# Automated, aseptic sampling with small-volume capacity from microbioreactors for cell therapy process analysis

**DOI:** 10.3389/fbioe.2025.1612648

**Published:** 2025-07-31

**Authors:** Zhi Xian Chan, Shruthi Pandi Chelvam, Wei-Xiang Sin, Denise Bei Lin Teo, Ahmad Amirul Bin Abdul Rahim, Ying Ying Wu, Dan Liu, Michael E. Birnbaum, Derrick Yong, Rajeev J. Ram

**Affiliations:** ^1^ Critical Analytics for Manufacturing Personalized Medicine, Singapore MIT Alliance for Research and Technology Centre, Singapore, Singapore; ^2^ Biomanufacturing Technology, Bioprocessing Technology Institute, Agency for Science, Technology and Research (A*STAR), Singapore, Singapore; ^3^ Department of Biological Engineering, Massachusetts Institute of Technology, Cambridge, MA, United States; ^4^ Agency for Science, Technology and Research (A*STAR), Singapore, Singapore; ^5^ Department of Biomedical Engineering, National University of Singapore, Singapore, Singapore; ^6^ Department of Electrical Engineering and Computer Science, Massachusetts Institute of Technology, Cambridge, MA, United States

**Keywords:** autosampler, aseptic, small-volume, car-t, bioreactor, microbioreactor, sampling

## Abstract

Current workflows in autologous cell therapy manufacturing are reliant on manual processes that are difficult to scale out to meet patient demands. High throughput bioreactor systems that enable multiple cultures to occur in parallel can address this need, but require good bioprocess monitoring workflows to produce good quality cell therapy products. Commercial sampling systems have thus been developed for better feedback control and monitoring capabilities. However, they are targeted towards large scale processes and often bioreactor specific, making them less robust for integration across different bioreactor scales and types, such as perfusion-capable microbioreactors which allows for greater process intensification. Here, an automated cell culture sampling system (Auto-CeSS) was developed to eliminate laborious manual sampling while minimizing sterility risks for cell therapy manufacturing processes. The system is aseptically integrated with a variety of bioreactors of different working volumes. This system can accurately and aseptically sample a minimum volume of 30 μL and can consistently perform periodic sampling of supernatant over a minimum interval of 15 min. We integrated Auto-CeSS with a 2 mL perfusion microbioreactor and a 8 mL gas-permeable well-plate for T cell culture, collecting 200 μL of supernatant samples daily for metabolite analysis. Comparison of the metabolic profiles of the samples collected via Auto-CeSS versus manual sampling revealed insignificant differences in metabolite levels, including glucose, lactate, glutamine, and glutamate. This report demonstrates the potential of Auto-CeSS as an at-line sampling platform in a real-time T cell production run to facilitate in-process culture monitoring.

## 1 Introduction

Cell-based therapies are a class of advanced therapy medicinal products (ATMPs) that are dramatically changing the landscape of regenerative medicine. (Chilmonczyk and Fedorov, 2021). In particular, Chimeric Antigen Receptor (CAR) T cell therapies are a form of cell-based immunotherapies that are revolutionizing the treatment of various types of cancer, especially of hematological malignancies such as B cell lymphomas, leukemias and multiple myeloma (Alnefaie et al., 2022). CAR T cell therapies that are in clinical use have demonstrated successful patient outcomes, but challenges remain in their engineering and manufacturing, including production scalability to serve larger patient populations, process control and reproducibility, as well as product consistency and variability (Trainor et al., 2023).

To address these manufacturing challenges, there is a recent push to utilize quality-by-design (QbD) principles to better understand how critical process parameters (CPPs) influence CAR T cell critical quality attributes (CQAs) (Lipsitz et al., 2016). Firstly, basic in-process monitoring of crucial metabolites in the culture medium can serve as surrogate markers for cell health and state during *ex vivo* T cell expansion. For example, glucose is a primary energy source that is required for proper T cell activation and T cell growth, (Palmer et al., 2015) whereas excessive levels of lactic acid in the culture medium can suppress T cell proliferation and function (Quinn et al., 2020). With rapid glucose consumption and lactate production during *ex vivo* T cell expansion following upregulation of aerobic glycolysis upon T cell activation, glucose and lactate levels in the culture medium can be closely monitored to track viable cell numbers in the culture vessel over time. (Grist et al., 2018; Gagliardi et al., 2019; Coeshott et al., 2019; Ludwig et al., 2020). Secondly, advanced process control of culture conditions can potentially be used to direct T cells towards desired phenotypes or functions. (Sudarsanam et al., 2022). Prior studies show that levels of metabolites such as glucose, lactate and glutamine can metabolically reprogram T cells and maintain cells in a less-differentiated state for enhanced efficacy and persistence (Shen et al., 2022; Feng et al., 2022; Cappabianca et al., 2024). These and other evidence suggest that the concentrations of substrates in the cell culture medium during CAR T cell manufacturing are potential CPPs that can fine-tune T cell metabolic fitness and differentiation status, and thus improve the consistency and quality of the cell therapy products. (Piscopo et al., 2018; Wiley Online Library, 2024). Central to the implementation of such metabolite monitoring and control strategies is the integration of process analytical technologies (PATs) into the CAR T cell manufacturing workflow. Ultimately, this can enable continuous quality verification, as well as provide early detection of out-of-specification products or batch failures, thereby improving process efficiency and product quality (Wiley Online Library, 2024).

Off-line or at-line PATs that are based on metabolite analysis of culture media require aliquots of spent media to be extracted from cultures, which is traditionally performed via manual methods. These manual methods usually involve the use of syringes or pipettes carried out by trained personnel. Due to differing levels of training and experience, the consistency of manual sampling is prone to operator variability and error, potentially compromising sterility and increasing the risk of contamination. (Musmann et al., 2024). Therefore, it is desirable that manual open-processes such as sampling be automated, which minimizes laborious and time-consuming procedures, reduces operator error, and ensures sterility. Faster process optimization with the help of automation also assures more consistent product quality, driving the process to be more regulatory-compliant. (Doulgkeroglou et al., 2020; Marques and Szita, 2017). Automated sampling operations are particularly beneficial when interfacing with automated perfusion bioreactors, which produce a continuous output of spent media from continuous media exchange for downstream analysis.

Several automated sampling systems including MAST (Merck, Germany) (MAST^®^ Autosampling So lution, 2025), SegFlow PS (Flownamics, United States) (Flownamics, 2025), Omnicoll Fraction Collector (LAMBDA Instruments GmbH, Switzerland) (OMNICOLL-fraction-collector-and-sampler, 2025; LAMBDA Laboratory Instruments, 2025), EasySampler 1210 (Mettler Toledo, United States) (reser ved MTII all rights, 2025), CliniMACS Formulation Unit (Miltenyi Biotec, Germany) have been used widely in cell therapy manufacturing. Since cell therapy products have limited starting material available for process and product development, (Bartel et al., 2015), it is crucial to keep sampling volumes at minimum to prioritize the final cell therapy product dose to be infused into the patient. (Iancu and Kandalaft, 2020) Most commercial sampling systems have a minimum sampling volume of 1 mL, which makes them incompatible for smaller scale operations. Thus, there is a need for developing small-volume automated sampling systems that are compatible with scaled-down microbioreactors, such as for the 2 mL microfluidic microbioreactor (Mobius Breez, MilliporeSigma), on which we have recently established a CAR T cell production process. (Sin et al., 2024). The perfusion-mode microbioreactor allowed for rapid reagent changes in the 2 mL growth chamber, enabling human primary T cells to be activated, transduced and expanded to high densities. In Ref (Sin et al., 2024), we demonstrated the production of viable anti-CD19 CAR T cells (specifically, more than 60 million CAR T cells from donor cells derived from patients with lymphoma and more than 200 million CAR T cells from healthy donors) – sufficient for a complete therapeutic dose. However, monitoring of critical metabolites within the microbioreactor required manual sampling of the perfusate.

Here, we present an Automated Cell Culture Sampling System (Auto-CeSS) as an aseptic, small-volume sampling platform that can be integrated with bioreactors for periodic sampling of cell culture supernatant. The system is capable of accurately sampling volumes as low as 30 μL and is able to sample at a 15 min interval. To demonstrate the robustness of the system, we have interfaced Auto-CeSS with the Mobius Breez microbioreactor and larger-scale bioreactors such as the 8 mL gas-permeable well-plate (G-Rex 10M-CS, Wilson Wolf) to sample 200 μL of cell culture supernatant from Days 2–12 of T cell culture for off-line metabolite monitoring. Metabolic monitoring is especially important for cell therapy products as cellular metabolism regulates cell health and differentiation, affecting the potency of the therapy (Ma et al., 2024). Comparable metabolite profiles were achieved for automatically extracted samples compared with manually extracted spent media. This system can potentially be further developed as an at-line sampling platform with integrated PATs, for automated sampling and measurements of cell culture bioprocesses.

## 2 Methods

### 2.1 The Auto-CeSS

**FIGURE 1:**
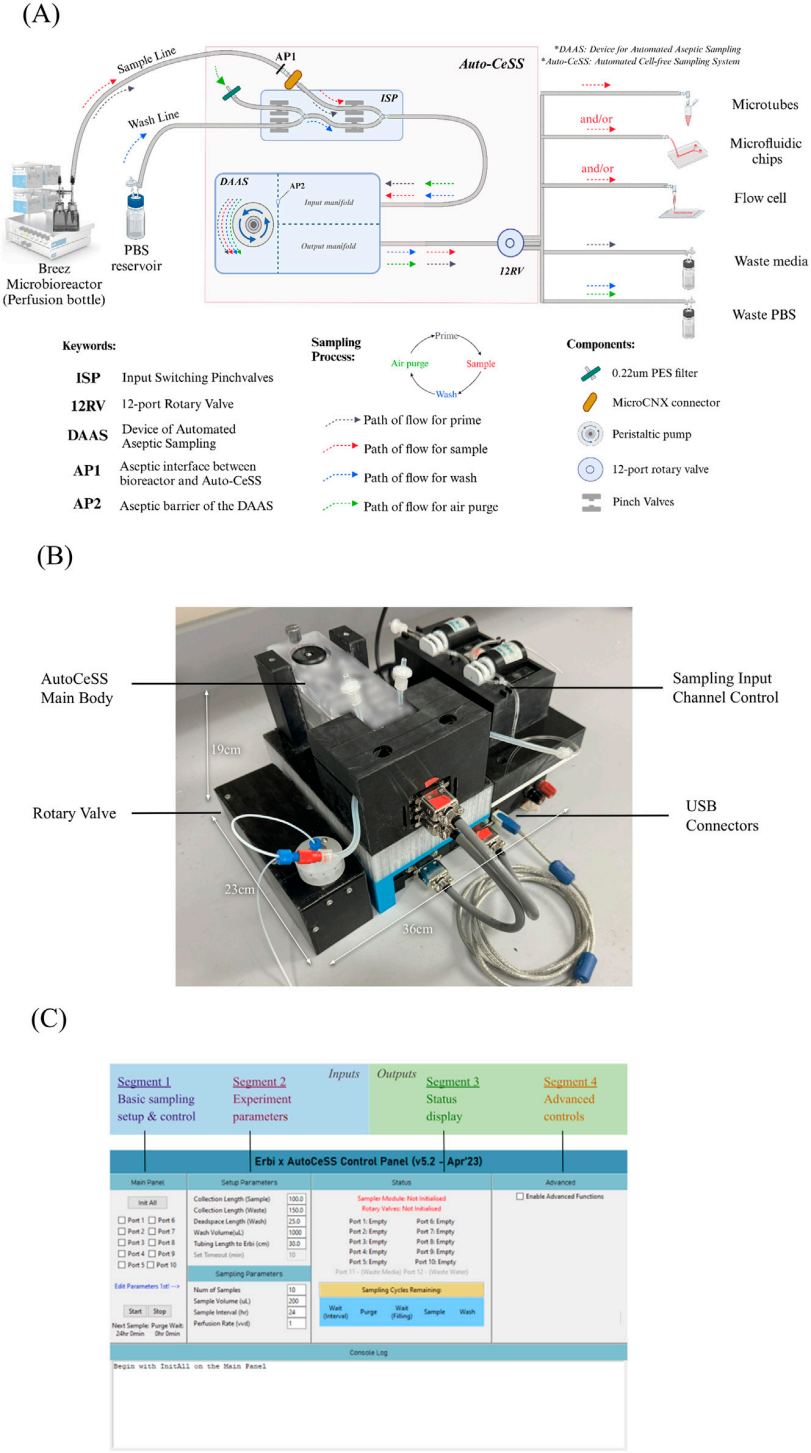
Overlay of the Auto-CeSS, including schematics and breakdown of components. **(A)** details the components of Auto-CeSS and the pathways of the prime, sample, wash and air purge steps which occur sequentially each time Auto-CeSS is used. The system has potential multiple downstream applications supporting off-line and at-line analysis. Peristaltic pump and motorized pinch valves drive the system, while the internal modules in the DAAS maintain a feedback system to the microcontroller for periodic sampling. Illustration created with BioRender.com. **(B)** details the real-life implementation of Auto-CeSS. The small footprint of this system compared to other commercial sampling systems allows for it to be mounted on a trolley to be conveniently moved around. **(C)** depicts the software control GUI developed for interface with the components and control of the whole sampling processes.

The Auto-CeSS seeks to interface between bioreactors of choice upstream and collection outlets of choice downstream. This system (Figure 1A) leverages the use of Device of Automated Aseptic Sampling (DAAS). (Dan et al., 2024). The sampler is regulated by a series of pinch valves and microfluidic-peristaltic pump to extract small volume samples from a bioreactor, with minimal dead volume, and requires the user to connect the required tubings into the input and output manifold respectively. Within the input manifold, the sampler maintains an aseptic barrier (AP2, Figure 1A) that ensures the sterility of segments upstream of the sampler. The input of the sampler is connected upstream to our Inlet Sampling Pinch valves module (ISP), whereby we control the input of 2 channels, a sample line and a wash line. The sample line is connected to a bioreactor via MicroCNX aseptic connectors (CPC) to establish another aseptic point (AP1, Figure 1A) between the upstream bioreactor and the downstream components of the sampler. The wash line is connected to a PBS *reservoir* bottle for wash and purge functionality. The ISP is responsible for determining whether the sample line or wash line is activated during system processes. The output of the sampler is connected to a 12-port rotary valve (AMF microfluidics) to enable multi-channel sample collection. 2 out of the 12 ports have been allocated for the collection of *Waste media and Waste PBS.*

The Auto-CeSS, additionally has 2 hardware modules (Figure 1A); namely, the ISP (upstream of sampler) for input channel switching and a 12-port rotary valve (downstream of sampler). This allows us to expand on the capabilities of the sampler, to include a wash and purge function to clean the system between sampling cycles and to also enable multi-channel sample collection. A comprehensive GUI has been developed to control each module of the Auto-CeSS with the use of customized software architecture (Supplementary Figures S1.1, 1.2) specific to each module. The implementation of these modules allows up to 10 samples to be collected without human intervention and for the wash and purge sequencing to occur automatically after each sampling round.

Prior studies have demonstrated use of the DAAS sampler with a specialized 50 mL culture vessel consisting of an expandable flask (Dan et al., 2024) from which cells have been sampled to determine cell count and cell viability. Here, we demonstrate integration of Auto-CeSS with small volume (2 mL) microfluidic bioreactors such as the perfusion-based Breez microbioreactor. We have also focused on sterile sampling of supernatants from perfusion cultures for metabolic analysis. This requires us to establish the independence of each extracted sample–in other words to develop protocols and hardware to minimize cross-talk between samples.

### 2.2 Hardware assembly of Auto-CeSS

A sterilization and assembly protocol is closely followed to set-up the Auto-CeSS to be sterile prior to use. Tubings used throughout the system are washed with 70% ethanol and connectors are wiped clean with 70% ethanol. 50 mL Duran laboratory bottles are used as *PBS reservoir*, *Waste media* and *Waste PBS* bottles and are fitted with 3-port GL45 screw caps (VICI). Subsequently, tubings, connectors, Duran bottles and screw caps are autoclaved at 121°C for 20 min and are placed in a drying oven overnight prior to assembly.

Parts of the sampler that cannot be autoclaved due to the material incompatibility are wiped thoroughly with 70% ethanol, dried and UV sterilized in the BioSafety Cabinet (BSC) for 30 min. Upon completion, the UV sterilized parts are assembled with the necessary autoclaved parts within a BSC. All air ports are fitted with 0.22 um PES filters to enable sterile air flow throughout the set-up. The completed set-up of Auto-CeSS is illustrated in Figure 1.

To interface the Auto-CeSS with the bioreactor, the sample line of Auto-CeSS is fitted with one-end of an autoclaved MicroCNX connector (CPC), while the other end of the autoclaved MicroCNX is attached to the sample port of the bioreactor in a BSC. The ends of the MicroCNX are sprayed with 70% ethanol and are connected aseptically at room environment, establishing connection.

### 2.3 T cell culture in 2 mL perfusion microbioreactor

Primary human PBMCs from healthy donors were obtained from a cryopreserved leukopak (Lonza). T cells were isolated using EasySep Human T Cell Isolation Kit (STEMCELL Technologies), and resuspended in AIM V Medium (Thermo Fisher Scientific) supplemented with 2% human male AB serum (Merck Sigma-Aldrich) and 100 IU/mL recombinant human IL-2 (Miltenyi Biotec). For activation, purified T cells at 1 million cells/mL were mixed with Dynabeads Human T-Expander CD3/CD28 (Thermo Fisher Scientific) at a 1:1 cell:bead ratio in a 25 mL tube, and 2 mL of cell-bead mixture was seeded into each microbioreactor cassette (Erbi Biosystems, MilliporeSigma). Two days after activation, perfusion was started at 1 vessel volume per day (vvd, 1 vessel volume = 2 mL) for the microbioreactor, and the perfusion flow rate was increased by 1 vvd every other day until a maximum of 4 vvd, with a constant culture volume of 2 mL. Cell-free medium samples were taken every ∼24 h by removal of ∼200 μL perfusate (accumulated over ∼2.4 h for 1 vvd, 1.2 h for 2 vvd, 48 min for 3 vvd, and 36 min for 4 vvd) from the perfusion output microtube within the perfusion output bottle, either using a syringe (manual sampling) or using Auto-CeSS (automatic sampling, see below). The microbioreactor cultures were controlled at the following set-points: temperature of 37°C, minimum CO_2_ levels of 5%, minimum dissolved O_2_ levels of 80% air saturation, and a pH of 7.40 ± 0.05. T cells were expanded for 12 days post-activation, with the microbioreactor operating in batch-mode for 2 days followed by perfusion-mode for 10 days.

### 2.4 Auto-CeSS integration with 2 mL perfusion microbioreactor

The Breez is an automated, perfusion based microbioreactor, which has a perfusion waste bottle containing a 1.5 mL perfusion output tube. The Breez permits a continuous in-flow and out-flow of media from a media bottle to the growth chamber and then to a perfusion output tube. The 1.5 mL tube starts to fill up with perfusate depending on the rate of perfusion, determined by the user. In the event that the tube fills up, perfusate overflows into the perfusion waste bottle. We integrated the Auto-CeSS with the perfusion output tube within the perfusion waste bottle, allowing for periodic sampling of the perfusate for metabolite monitoring of culture.

Initially, the tubing leading into the 1.5 mL perfusion output tube of the perfusion waste bottle was fitted with a needle-less connector allowing for sampling of the perfusate using a syringe. For us to enable real-time perfusate collection via Auto-CeSS, we modified the configuration of the tubing by replacing the needle-less connector to one end of the MicroCNX connector. This end was aseptically connected to its pair at the sample line of Auto-CeSS, establishing flow from the perfusion waste bottle to downstream microtubes via Auto-CeSS. An initial extraction of the perfusate is done to clear the contents of the microtube in the perfusion waste bottle. After waiting for a set period of time (depending on the perfusion rate), 200 μL fresh perfusate is collected for metabolite analysis, allowing for more accurate representation of the sampling time-point.

The Auto-CeSS was positioned on a lab bench next to the Breez and its associated bottle racks. To collect perfusate samples from days 2–12, the downstream of the sampler was fitted with a 12-port rotary valve (Advanced MicroFluidics) connected to reservoir holder racks (Elveflow) fitted with 10 microtubes for sample collection. Each day’s perfusate sample corresponds to one microtube between Ports 1-10. The microtube in Port 1 is replaced with a fresh tube for the last day of sampling (Day 12). Ports 11 and 12 are used as *Waste media* and *Waste PBS* respectively. A commercial mini-fridge set at a temperature of 4°C was used to house the reservoir holder racks and its microtubes, allowing for samples to be held cold over the course of the experiment. At the end of the culture (Day 12), microtubes containing samples from days 2–12 were removed from the fridge and frozen at −80°C. The collected perfusate samples were then measured using the Cedex BioAnalyzer (Roche CustomBiotech).

As shown in Supplementary Figures S3, T cell phenotype was analyzed by flow cytometry. Almost all the cells were CD3^+^ T cells. A high percentage of T cells expressed the CAR, and the proportions of CD4^+^ and CD8^+^ T cells, naïve, central memory (CM), effector memory (EM), and terminal effector (TEMRA) T cells were as shown, indicating that the majority of the cells were of the naïve phenotype. Incorporation of Auto-CeSS did not affect T cell phenotype within the Breez microbioreactor.

### 2.5 T cell culture in 8 mL gas-permeable well-plate

The G-Rex 10M-CS is a closed system, sterile fluid path device, and is part of the M series devices, which have a tall medium height (large medium capacity) that do not require medium exchanges. 10 million purified T cells obtained as described above were activated with Dynabeads Human T-Expander CD3/CD28 (Thermo Fisher Scientific) at a 1:1 cell:bead ratio and seeded in 100 mL AIM V Medium (Thermo Fisher Scientific) supplemented with 2% human male AB serum (Merck Sigma-Aldrich) and 100 IU/mL recombinant human IL-2 (Miltenyi Biotec) in a G-Rex 10M-CS. Cell-free medium samples were taken every ∼24 h by removal of 200 μL culture supernatant from the sample port line, either using a syringe (manual sampling) or using Auto-CeSS (automatic sampling, see below). The G-Rex 10M-CS was placed in a standard 37°C, 5% CO_2_ incubator. T cells were expanded for 12 days post-activation, with no medium exchange.

### 2.6 Auto-CeSS integration with 8 mL gas-permeable well-plate

We interfaced the Auto-CeSS with the sample port of the G-Rex 10M-CS, which terminates approximately 50% of the way into the vessel. The Clave™ needleless septum that was originally fitted at the end of the sample port was replaced with additional tubing with a y-connector. One side of the y-connector was connected to a needle-less port to facilitate manual sampling with a syringe. The other end of the y-connector was fitted with a MicroCNX connector to be interfaced with the other end connected to the sample line of Auto-CeSS. This set-up was placed in an incubator, with tubing connected to the MicroCNX running through the access port at the back of the incubator.

The Auto-CeSS was mounted on a trolley and positioned next to the incubator. The ends of MicroCNX connectors were aseptically connected. 200 μL of cell culture supernatant was sampled daily from Days 2–12 and frozen immediately at -80°C. Cell counts were measured using Acridine Orange (AO) and Propidium Iodide (PI) and/or Trypan Blue (TB) staining on a CellDrop Automated Cell Counter (DeNovix).

### 2.7 Cell counts

Cell counts were measured using Acridine Orange (AO) and Propidium Iodide (PI) and/or Trypan Blue (TB) staining on a CellDrop Automated Cell Counter (DeNovix).

### 2.8 Cellular medium metabolite analysis

Metabolites in cell medium, including glucose, lactate, ammonia, glutamine and glutamate were measured on a Cedex BioAnalyzer (Roche CustomBiotech) according to manufacturer instructions.

### 2.9 Culture-based sterility test

The Auto-CeSS assembled as per the protocol stated in Section “Auto-CeSS integration with 2 mL Perfusion Microbioreactor” was interfaced with the G-Rex 10M-CS with 10 million purified T cells as mentioned above in Section “T cell culture in 8 mL Gas-Permeable Well-Plate”. 1000 μL samples were extracted in triplicates from the G-Rex at a 45 min interval and dispensed into Ports 1-3 (outside the BSC). Upon completion of sampling, 1000 μL of PBS was used to wash the system, which was channeled into the PBS waste reservoir. The microtube at Port 1 was replaced with a new microtube (in room environment), in which Sample 4 was collected. Other samples that were collected include aliquots of stock PBS and stock AIM V media taken directly from the bottle provided by the supplier. AIM V media aliquots were taken from the G-Rex at the start and end of the experiment. 1000 μL of PBS samples were taken from the PBS reservoir at the start and end of the experiment. PBS aliquot was extracted from the Waste PBS bottle at the end of the experiment.

100 μL of collected samples were added to 5 mL of TSB broth or 10 mL of thioglycollate and incubated at 22.5°C or 32.5°C respectively, with shaking at 150 rpm for samples inoculated in TSB broth. Samples were visually inspected for turbidity on Days 4, 7, 10 and 14. Tubes were discarded on Day 14.

*Bacillus Subtilis* ATCC 6633 was inoculated in TSB broth, while *Staphylococcus Aureus* ATCC 6538 was inoculated in thioglycollate as positive controls. Negative control tubes had broth only, with no additional inoculation.

## 3 Results

### 3.1 Development of the Auto-CeSS

Building on the DAAS, the cornerstone of the Auto-CeSS workflow lies in the implementation of the sampling control process, downstream hardware additions such as the rotary valve and integrated software controls, which provides potential downstream attachments such as:

1. Fitting outlets of rotary valve with microtubes for sample collection and off-line analysis.
2. Connecting to microfluidic chips for micro-scale in-line and at-line culture analysis.
3. Connecting to optical cuvettes for integration with PATs for real-time, at-line culture monitoring.

### 3.2 Hardware

In Figure 1A, the Auto-CeSS demonstrates a use case of sample collection from a microbioreactor. To initiate the priming step, culture media is pulled from the bioreactor towards the main sampling body. Time-based switching of pinch valves allows for a 100 μL sample aliquot to be created and pushed towards the outlet of the sampler and subsequently dispensed into the *Waste media* bottle through the rotary valve. This aliquot primes the tubing with culture media.

Thereafter, the sampling cycle starts with culture media being extracted from the bioreactor till the aseptic barrier in AP2. Switching of pinch valves allow for a sample bubble of desired volume to be created and pushed towards the outlet and subsequently dispensed into microtubes/microfluidic chips/flow cells connected to the rotary valve ports. Afterwhich, the remaining fluid within the tubings till point AP2 is reversed back into the bioreactor resulting in no dead volume.

Post-sampling, a wash cycle is carried out where PBS is drawn from the *PBS reservoir* and flushed through the tubings through the outlet of the sampler, into the *Waste PBS* bottle. An air purge follows after the washing of PBS). Table 1 further elaborates the features of the different phases the Auto-CeSS runs through during a sample and wash and purge cycle.

**TABLE 1:** Operational mechanisms that was developed for the Auto-CeSS which maintains the whole working process and allows for robust sampling to occur. The basic operations involve sampling, washing and priming. These are tied together through the main code interacting with the in-built microcontroller, allowing for further implementation of time-based contingencies. This allows the Auto-CeSS to account for software/hardware failure, whereby the whole process is monitored and can be automatically halted should failure be detected, prompting the user of any failure/manual override requirements.

Phase	Auto-CeSS features	Rationale
Sample cycle	Functionally closed liquid transfers	To prevent contamination of samples
	Pushing back of cell culture media from sampler into bioreactor, post sampling	To enable zero dead-volume sampling, prevent wastage of media
	Aseptic barrier	To ensure sterility between bioreactor and sampler, ensuring drawn samples remain sterile despite sterility condition of outlet
Wash cycle	Intermediate wash cycles with PBS between sampling cycles	To prevent cell debris (if any) from clogging the system. To remove remnants of previous samples, ensuring no cross-talk between consecutive samples
	Air purge post wash cycle	To ensure that tubings are dry and ready for next round of sampling
Priming	50 uL aliquot of culture is pulled from the bioreactor to run through tubings of sampler	Helps to get rid of any PBS remnants left behind after wash cycle ensuring minimal dilution of samples
Sequential Control (Arduino Microcontroller)	Placement of tube sensors at inlet and outlet of sampler, microcontroller hub	Main driver of all protocols for the Auto-CeSS, acts as the main control for accurate volume-based sampling and sequence of operations
Contingencies	Time-based failsafe overrides, advanced hardware controls and emergency stop button on GUI	To prevent occurrences of sampler indefinitely running, as well as manual override for additional control

### 3.3 Software

The main controller of the sampler consists of an Arduino MEGA AT2560 and connected via USB to the computer. The main driver code is coded with Python, and serial communication is established via COM port to the Arduino MEGA for callouts. The callouts enable the full sampling/wash and purge procedures. Additional capabilities include individual control of each pinch valve as well as rotary valve direction and speed, which are used for troubleshooting and advanced functionality development.

The rotary valve module is also connected via USB to the computer and runs over Serial to RS-485 connection for callouts.

A Graphical User Interface (GUI) is constructed using Python as the main controller for the system (Figure 1C). The GUI is made agnostic and can be used for implementation with the Breez, G-Rex and other bioreactors if required. The main prerequisite occurs in Segment 1 and Segment 2, whereby the GUI requires user input of critical experimental parameters before the experiment begins. This enables a one-click sampling process, for which the user need not return to the system until the completion of the whole cycle. The GUI is also designed with the robustness to account for updating of parameters during the experiment.

### 3.4 Validation studies of the Auto-CeSS

#### 3.4.1 Minimum sampling volume

To determine the minimum sample volume of the sampler, different volumes of water (10, 20, 30, 40, and 90 μL) were sampled from a water reservoir at 30 min intervals, and the mass of the extracted water was measured. A total of 10 trials were performed for each sample volume. Supplementary Table S1 summarizes the mean and standard deviation obtained for each intended sample volume. Statistical analyses indicate that the sampler can accurately sample a minimum of 30 μL (p > 0.05, two-tailed t-test), which is lower than the minimum sampling volumes of existing autosamplers for bioreactors.

#### 3.4.2 Optimization of “wash and purge” functionality

**FIGURE 2:**
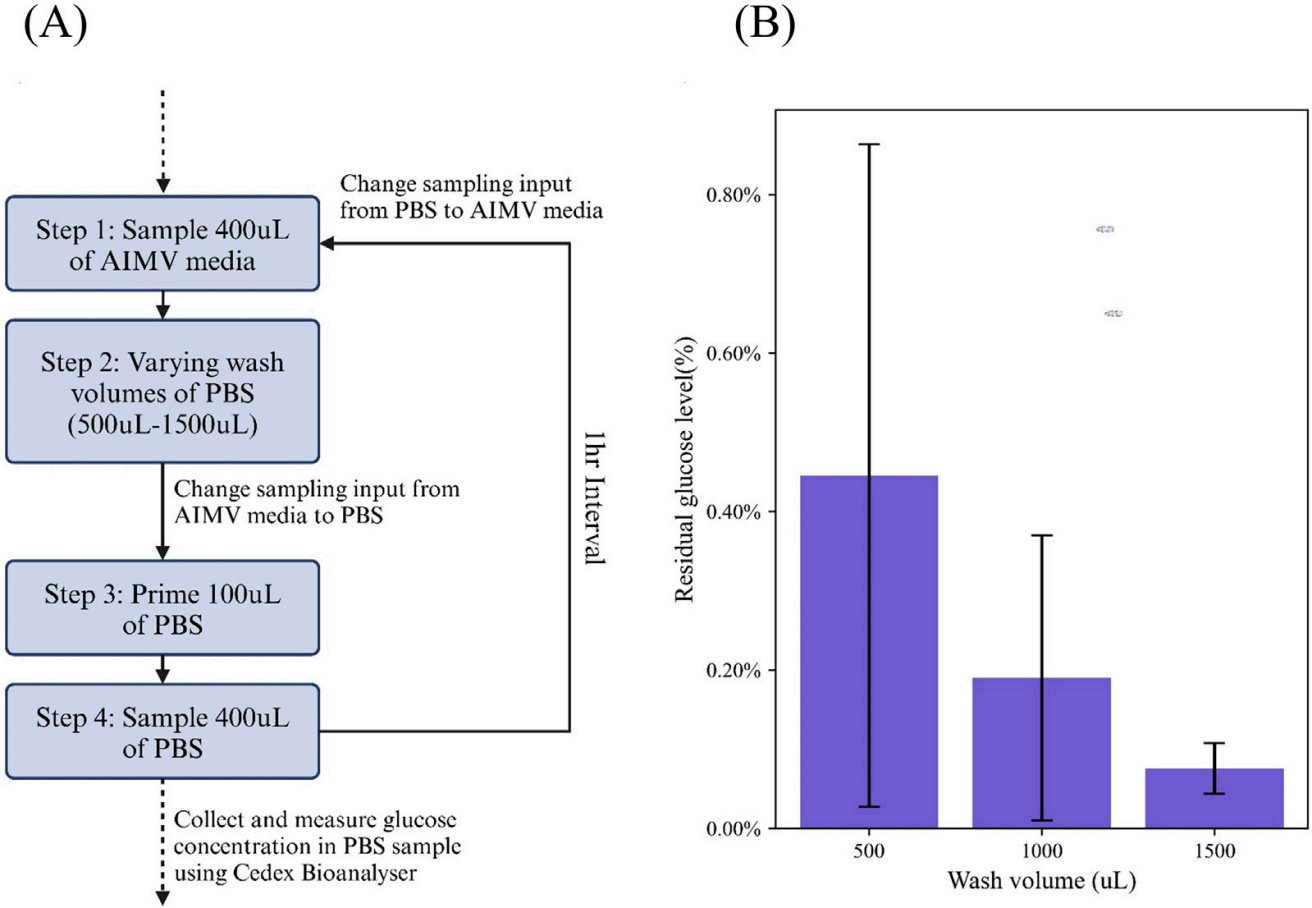
Experimental design to determine the ideal volume of PBS required for a through wash and purge cycle. **(A)** Workflow carried out in this study **(B)** Results of the residual glucose level based on the concentration of glucose in an initial 400uL AIMV sample, post a 500uL, 1000uL and 1500uL PBS wash.

To determine the ideal PBS volume for wash, we performed a test which varies the wash volume used between each sample (Figure 2). The system first sampled 400 μL AIMV culture media, then washed with 500 μL, 1000 μL or 1500 μL of PBS. Lastly, the sample inlet was switched from AIM V culture media to PBS, and a priming step of 100 μL of PBS was carried out, followed by 400 μL of PBS sample which was collected at the outlet of the sampler. PBS samples were collected in triplicates, with an hour wait interval. Collected samples were measured using the Cedex Bio Analyzer for traces of glucose (Figure 2B). The glucose levels in the collected PBS sample were expressed as a percentage of the glucose levels normally found in AIMV culture media (100% = 16.3 mmol/L).

The data shows that ≥1000 μL PBS of wash is required to achieve under 0.5% sample carry-over. This ensures a sufficient wash of the system, eliminating any residue from the previous sample to interfere with the next sample. Alternatively, the user may choose to set the acceptable threshold of sample carry-over higher or lower and vary the wash volume, accordingly, depending on the stringency of the experimental requirements. Nevertheless, based on a cut-off of <0.5% sample carry-over, we decided on 1000 μL as the wash volume in our protocols.

#### 3.4.3 Sampling interval

**FIGURE 3:**
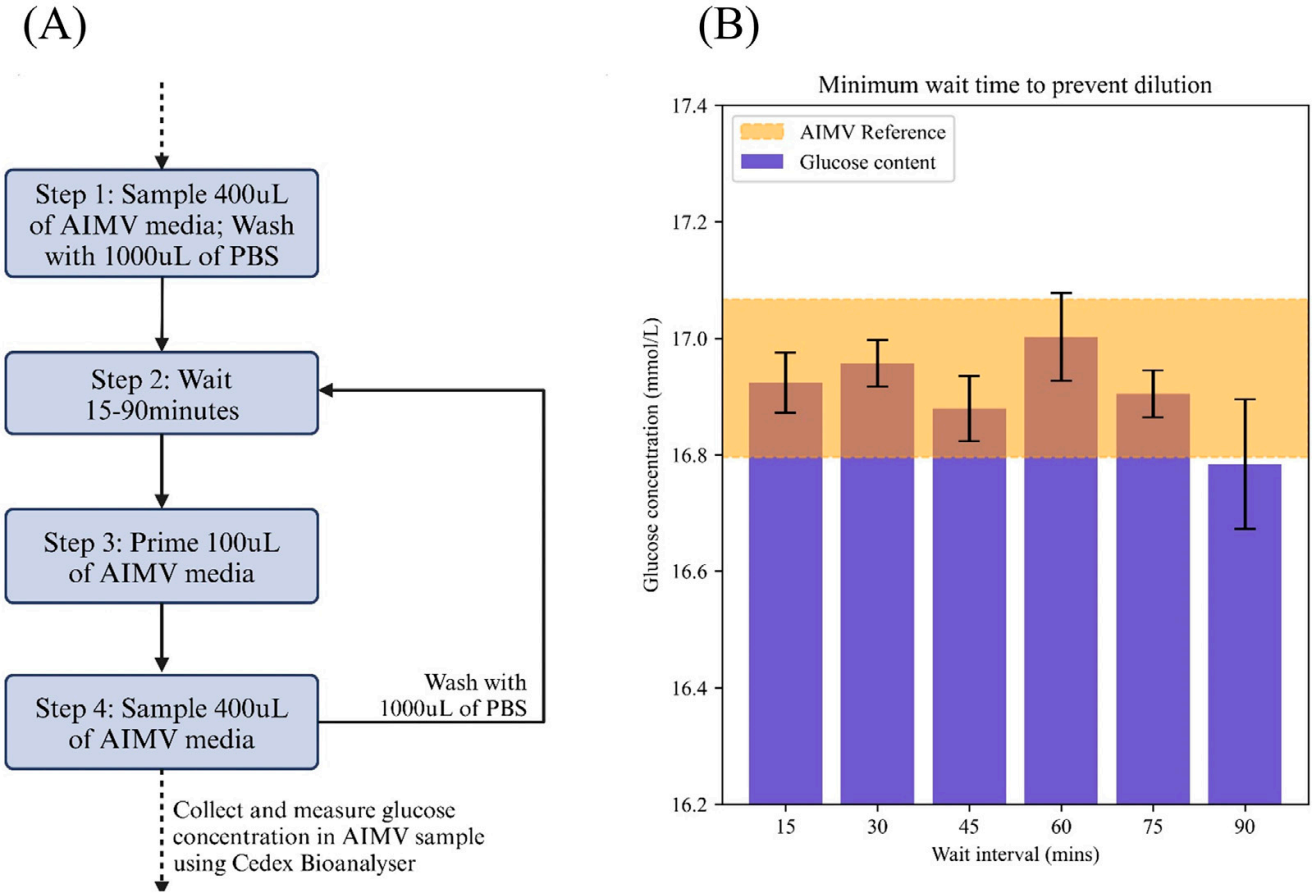
Experimental design to determine the optimal wait intervals between samples to prevent dilution of samples due to PBS. **(A)** Workflow used in this study **(B)** Glucose concentration of samples collected after 15–90 min wait intervals and compared against reference sample concentrations. The reference value for AIMV was taken 3 times and a range was generated based on one-sigma allowance (16.932 ± 0.135). Each sample interval box-plot is representative of each wait interval measurement taken in triplicates. Wait interval was kept to a minimum of 15 min.

Due to the nature of PBS based washing in between sampling cycles, residual unevaporated PBS may cause dilution onto the samples collected. With the implementation of the pre-purge functionality, whereby 100 μL of the intended sample was passed through the system before conducting the actual sample, we were able to obtain consistent after a minimum sampling interval of 15 min (Figure 3). We performed a reference measurement of the stock AIM V media and compared it against triplicates of the system-collected AIM V samples. All samples were measured using the Cedex Bio Analyzer for glucose concentration levels (Figure 3B). The reference value was presented as a range using the standard deviation for comparison with collected samples.

#### 3.4.4 Sterility testing

Results of the growth-based sterility test of samples collected from Auto-CeSS and reagents involved are summarized in Table 2. Images of sterility studies are illustrated in Supplementary Figure S2.

**TABLE 2:** Results from sterility testing procedures based on collection using the Auto-CeSS with the G-Rex. Sample ports 1-3 were set up in a fully sterile BSC with corresponding samples 1-3 flowing through the respective port numbers. Sample 4 was collected by reusing the sample port 1 at the end of the sampling sequence, and underwent a tube change which exposed the outlet port to non-sterile environment. Additional AIMV and PBS reference samples were collected at the start and end of the experiment. All samples (inclusive of positive and negative control samples) were inoculated in TSB and FTM broths and incubated. Only the positive control samples presented with turbidity on Day 4, while all other samples remained clear.

Samples	TSB broth	Thioglycollate broth
Negative control (TSB broth)	Clear	
Negative control (Thioglycollate broth)		
Samples 1,2,3		
Sample 4 (Tube Change)		
Stock PBS		
Stock AIMV media		
AIM V in G-Rex at start of experiment		
AIM V in G-Rex at end of experiment		
PBS in *PBS reservoir* at start of experiment		
PBS in *Waste PBS*		
*Bacillus Subtilis ATCC* 6633	Turbidity observed on Day 4	-
*Staphylococcus Aureus ATCC 6538*	-	Turbidity observed on Day 4

The sterility studies verified that the assembly protocol of the Auto-CeSS (*Section “Hardware assembly of Auto-CeSS”)* maintained the aseptic integrity of the system. Furthermore, Auto-Cess did not introduce any contamination in the system, as there was no turbidity observed in *Waste PBS* and AIM V samples taken at the end of the experiment. Samples 1-3 were deemed sterile, giving confidence that the Auto-CeSS was able to sample aseptically whilst maintaining the sterility of the culture. Despite the change of the microtube in Port 1 being done in a non-sterile environment, Sample 4 was reported as sterile. This confirmed that the presence of the aseptic barrier of the system maintained upstream (from bioreactor till sampler), ensures that dispensed samples remain sterile despite the collection point being exposed to traces of contamination.

### 3.5 Integration with breez and G-Rex 10M-CS

#### 3.5.1 Accuracy of sampling volumes

**FIGURE 4:**
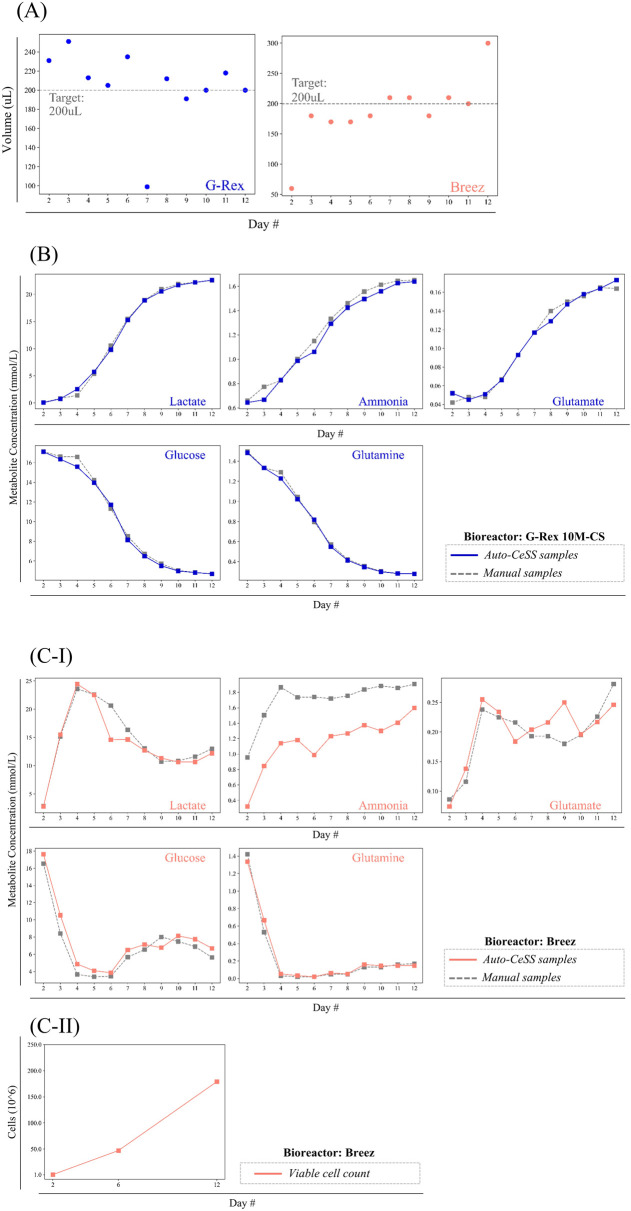
Quantitative data comparing supernatant samples collected with the Auto-CeSS against manual extraction methods across 2 different cell culture bioreactors. **(A)** Volume of samples collected by the Auto-CeSS during runs with the G-Rex 10M-CS(blue) and Breez (pink). Sample volumes were compared with the daily sample volume target of 200uL. **(B,C)** Collated the metabolite measurement from runs where Auto-CeSS was integrated with the **(B)** Breez and **(C)** G-Rex 10M-CS. Samples were collected daily from day 2–12, with 5 key metabolite indicators being measured using the Cedex Bioanalyser. The automated sample collected via the Auto-CeSS (blue/pink) is coupled with a manual sample (gray) for each data point, with a maximum interval of 5 min between the corresponding samples. Cell counts were conducted at day 2, 6, and 12 for the Breez (c-ii).

The Auto-CeSS was set to sample 200 μL of cell culture media when integrated with the Breez and G-Rex respectively. Figure 4A illustrates the performance of the sampler, over a 11-day period (Days 2–12 of culture) in accurately sampling 200 μL for both the Breez and the G-Rex. Samples taken from the Breez had a mean volume of 190.0 μL and standard deviation of 16.3 μL, while samples taken from the G-Rex had a mean volume of 207.9 μL and standard deviation of 37.2 μL.

There were 3 outliers identified, namely, on Days 2 and 12 of the Breez run, and on Day 7 of the G-Rex run. For the Breez run, Day 2 had a significantly lower sample volume collected due to low volume of perfusate dispensed into the perfusion waste tube arising from the Breez bioreactor (rather than an error on the part of Auto-CeSS). An abnormally high sample volume was recorded on Day 12 due to a power trip that occurred while sampling, which disconnected the Auto-CeSS mainboard from the control laptop. Day 7 of the G-Rex run experienced a low sample volume attributed to an air-bubble that was introduced into the Auto-CeSS system during the priming step, leading to the premature starting of the timer in the sensor.

#### 3.5.2 Metabolite monitoring

We previously monitored the metabolic state of T cells in culture within the Breez microbioreactor by daily manual sampling, withdrawing 200 μL of cell-free perfusate in the perfusion output tube using a syringe connected to the needle-less port, and then measuring metabolite levels off-line using a Cedex Bio Analyzer. (Sin et al., 2024). Prior experiments done in our lab involve daily manual sampling of 200 μL of cell culture media from the Breez and G-Rex bioreactors and were frozen immediately (−80°C). Samples were subsequently thawed to measure the concentrations of metabolites of interest via the Cedex Bioanalyzer. Figures 4B,C summarizes the metabolite concentrations of each sample collected via Auto-CeSS and manual sampling upon integration with G-Rex 10M-CS and Breez bioreactors. Cell counts were conducted for the G-Rex experiment during harvest on Day 12, resulting in a count of 62.6 million cells. Cell counts for the Breez microbioreactor (Figures 4C–II) were conducted through manual sampling directly from the culture vessel, and recorded a final harvest of 227 million cells.1

Upon integration of Auto-CeSS with the G-Rex, samples were frozen immediately (−80°C) while samples from the Breez experiments were stored in a 4°C refrigerator till the end of the CAR T cell production run (Day 12) before being stored at −80°C. To assess whether the Auto-CeSS performs comparably to manual sampling and whether the cooling of samples affects the measured metabolite levels in the samples, we examined the glucose and lactate levels in samples collected manually or via Auto-CeSS. Glucose and lactate concentrations in manual and Auto-CeSS extracted samples from the G-Rex were largely similar, indicating that sample collection via Auto-CeSS retained similar measured metabolic profiles as manual sampling.

Metabolite plots of G-Rex culture samples reflected a clean S-curve whereas plots of the Breez samples had asymmetric peaks present. This is likely due to the different mode of cultures between both bioreactors. For the G-Rex, a batch culture was performed with no medium exchange resulting in depletion of nutrients and waste products accumulation over time. The Breez had a perfusion culture with continuous medium exchange from Day 2. Thus, nutrients were continuously replenished while waste products were continuously removed.

Differences in glucose and lactate levels in the samples taken manually versus the samples taken using Auto-CeSS were slightly larger for the Breez experiment compared to the G-Rex experiment. One possible reason for this could be that manual and Auto-CeSS samples were taken from different growth chambers or “pods” run in parallel on the Breez, as opposed to manual and Auto-CeSS samples being taken from the same culture vessel for the G-Rex experiment. In other words, for the Breez experiments, T cells from the same donor were expanded in two separate “pods” or modules of the Breez microbioreactor, and manual sampling was performed for one pod, while the other pod was connected to the Auto-CeSS for automatic sampling. Nevertheless, despite some expected inter-sample variability attributed to the independent runs in two separate pods, differences in glucose and lactate levels were relatively subtle between the two sampling methods, indicating that samples derived from manual and Auto-CeSS sampling resulted in comparable metabolite profiles.

Metabolite plots of G-Rex culture samples reflected a clean S-curve whereas plots of the Breez samples had asymmetric peaks present. We hypothesize that this could be due to the variation in mode of cultures between both bioreactors. For the G-Rex, a batch culture was performed with no medium exchange resulting in nutrient consumption and waste products accumulation over time. The Breez had a perfusion culture with continuous medium exchange from Day 2. Thus, nutrients were continuously replenished while waste products were continuously removed.

Interestingly, comparison of ammonia levels for the manual and Auto-CeSS G-Rex experiments were comparable to each other, as both samples were immediately stored at −80°C after daily sampling. However, for the Breez experiments, the samples extracted via manual sampling were frozen immediately at −80°C while the samples pulled via Auto-CeSS were kept in the refrigerator at 4°C till Day 12 before freezing all the samples together at −80°C on Day 12. Ammonia levels were significantly lower in the samples kept in the refrigerator compared to the samples that were immediately frozen. For the Auto-CeSS integrated Breez run, the microtubes were fitted into a sample collection rack that contains a sterile air-port to enable fluids to flow in smoothly. Thus, it is possible for ammonia to evaporate over the duration it was left in the refrigerator. This demonstrates that storage temperature should be taken into consideration during sampling, as it can influence certain metabolite levels in the sample, depending on the type of metabolite.

## 4 Discussion

Several commercial automated sampling systems have been developed in recent years, such as the MAST (MAST^®^ Autosampling So lution, 2025), SegFlow PS (Flownamics, 2025), Omnicoll Fraction Collector (OMNICOLL-fraction-collector-and-sampler, 2025; LAMBDA Laboratory Instruments, 2025) and EasySampler (reser ved MTII all rights, 2025) (Table 3). Many of these systems are expensive resulting in limited adoption by many laboratories. (Efromson et al., 2021). In addition, many of these systems are not capable of sampling 200 μL and below, making them incompatible with the sampling volume requirements (50–200 μL) of small-scale bioreactors, such as a 2 mL microbioreactor process that we recently established for cell therapy manufacturing. In this work, we demonstrate that the Auto-CeSS, an automated sampling system that we developed, can be used as an at-line sampling platform for miniaturized perfusion-based microbioreactors (Breez) as well as for larger-scale fed-batch bioreactors (G-Rex). We successfully integrated Auto-CeSS with the G-Rex and Breez to periodically sample cell culture supernatant for downstream metabolite analyses over the course of a 12-day CAR-T cell production run, simulating real-world cell culture sampling scenarios.

**TABLE 3:** Summary of the commercially available devices for sample extraction in the cell therapy manufacturing environment, providing a broad overview over each sampling system’s features and capabilities in comparison with the Auto-CeSS.

Sampling device	Omnicoll fraction collector	MAST	SegFlow PS	EasySampler 1210	CliniMACs formulation unit	Auto-CeSS
Source	Lambda Instruments	Millipore Sigma	Flownamics	Mettler Toledo	Miltenyi Biotec	This study
Minimum sampling volume (mL)	0.05	SP100: 10.0 SP200: 1.0	0.25	0.2	30.0	0.03
Sterility	Not sterile	Aseptic	Aseptic	Aseptic	Aseptic	Aseptic
Extent of automation	Remote-controlled to control sample collection mode and parameters	GUI to schedule and monitor sampling cycles and analytical tests, review data	Integrated with FlowWeb Webserver	Integrated with EasyMax Chemical Synthesis reactors	Integrated with Prodigy bioreactor and single-use tubing set	Agnostic GUI to schedule and monitor sampling cycles for multiple bioreactor types
No. of upstream inputs	1–20	1–10	1–16	1	1	1
No of downstream outputs	1–288	1–4	1–4	1–12	1–4	1–10
Footprint (inches)	13.4 × 19.3	SP100: 4.25 × 3 × 3.5 SP200: 2.17 × 3.2 × 1.8	12.6 × 6.6 × 4.24	19.6 × 18.3 × 12.2	24.6 × 3.5 × 3.3	14.2 × 9.1 × 7.5
Wash protocol to minimize sample mixing	None, same pathway used for all samples	Yes, alcohol based flushing of system/tubes	Yes, washing solution for delivery line	No, cleaning procedure at the start and end of experiment	No, tubing set and sample collection bag is single use	Yes, PBS based wash in-between each sampling run

The Auto-CeSS is capable of sampling smaller volumes than most commercial automated sampling systems (as low as 30 μL) in an aseptic manner. Using the Auto-CeSS removes the need to transfer the G-Rex cell culture vessel from the incubator to the BSC for sampling and enables automated sampling from the Breez in a benchtop environment outside of a BSC. This reduction in manual, open operations help to reduce the risk of contamination and minimize the disturbance of the bioreactor environment. With aseptic set up and connection of the Auto-CeSS to bioreactors, we were able to obtain sterile samples, as shown by our sterility tests, despite automated sampling outside of a BSC in an academic tissue culture room facility. Auto-CeSS has a smaller footprint and lower cost to manufacture (∼$10,000 USD), making it more attractive for adoption by laboratories. However, while other sampling systems have a higher throughput in terms of the number of bioreactors that they can be interfaced with, the current Auto-CeSS prototype can sample at multiple time points but only from a single bioreactor. Future modifications of the Auto-CeSS, such as by adding a rotary valve upstream of the sampler, could allow the system to be connected to more bioreactors in parallel, enabling high-throughput sampling.

Despite having different culture volumes, both the Breez and the G-Rex bioreactors have a sample port that can be connected to an automated sampling system. However, for culture vessels without a sample port, such as T-flasks, a sample cap can be designed and fitted with the culture vessel to connect with the Auto-CeSS. In this way, the Auto-CeSS can interface with different types of bioreactors, thus exemplifying the agnostic capability of the system. Similarly, downstream of the sampler, we have interfaced the Auto-CeSS with a rotary valve that enabled multi-port sampling in this paper. In ongoing and future work, we are exploring the integration of Auto-CeSS with other components and PATs for various applications. For example, the Auto-CeSS can potentially be integrated with an optical flow cell for Raman spectroscopy (Baradez et al., 2018) and UV-Absorbance spectroscopy. (Chelvam et al., 2022). This would enable real-time, continuous metabolite monitoring of the cell culture, which could ultimately aid in the development of closed-loop feedback process control strategies in adaptive manufacturing protocols. (Liu et al., 2021; Hewitt et al., 2021). In-line analytical tools such as broad-spectrum analyzers like Raman spectroscopy require intensive calibration of sensors and reliance on pre-existing training datasets for intensive data calibration (Baradez et al., 2018; Marienberg et al., 2024). The Auto-CeSS for at-line or off-line analysis of medium samples can be used for benchmarking of these in-line measurements with current gold standard analyzers such as the Cedex or BioFlex metabolite analyzers.

One limitation of our system is the need for proper calibration of the pinch valves prior to the start of the experiment. There were instances where the uncalibrated pinch valves had failed to ensure proper flow control during the run, causing inaccuracies in sampling due to the pulsating flow and unintended mixing of reagents. It is therefore important to perform an initial calibration of the setup via the advanced user controls panel in the GUI to test the pinching capabilities of the valves.

Additionally, a 12V, 1A DC power supply and a direct USB connection is recommended for the Auto-CeSS for communication with the host computer. Not overloading multiple USB connections to a single USB port ensures that the USB connection is able to draw adequate power for smooth communication between the host computer and the microcontroller during operation. This will eliminate any inconsistencies in the pinch valve switching that might cause additional air bubbles to be introduced into the system, leading to sample volume loss or faulty sample and/or wash cycles. To minimize the sampling errors due to presence of air bubbles, we have made some adjustments on the software to reduce the sensitivity of the sensors to air bubbles. This reduces the chances of premature triggering of the sensor in response to presence of bubbles. Additionally, we have also improved the power stability of the system to reduce disconnection of Auto-CeSS from the control laptop. As an additional layer of checks, an *initialization* protocol has been implemented, before actual usage, to verify that all pinch valves are working as intended. Should the *initialization* protocol not pass, users can then perform troubleshooting by checking on the power supply connections and calibrating the valves.

Another limitation of the system is the absence of a cell debris filter. In the event that the cell culture has a high level of suspended cell debris, sampling the media from such cultures could lead to cell debris clogging the tubes, thereby impacting the performance of the sampler. Thus, there could be a need to implement a low retention, low dead-volume filter to minimize cell debris entering the sampler or the rotary valve. Alternatively, stronger solvents such as Isopropyl Alcohol (IPA) could be explored as a wash solution, instead of PBS.

As the Auto-CeSS is a proof-of-concept based sampling system, the next steps to meet Good Manufacturing Practices (GMP) compliance standards for use in the Cell and Gene Therapy (CGT) manufacturing pipelines include material selection and certifications, certifications of the device manufacturing process, modifications to the protocol for changing the collection microtubes, and enterprise-level updates to the software controlling the system. At this stage in development, the Auto-CeSS is currently suitable for use in Grade B or C cleanroom environment based on reported bacterial ingression studies (Dan et al., 2024). Future work to enhance the GMP readiness of the Auto-CeSS should include material evaluation of the different components. For instance, the plastic components in the Auto-CeSS need to meet USP Plastic Class VI requirements for biological reactivity, of which demonstrates the materials to be of low toxicity, low irritation and low infection rates via multiple tests. (Lin-Gibson et al., 2017; Addressing Particulates, 2018; US Pharmacopeia USP, 2025). We would also work on validation of cleaning protocols for essential reusable components such as the rotary valve, supported by risk-based frequency and effectiveness assessments. Washing and priming procedures can also be further optimized and line clearance protocols should be implemented to verify the removal of previous materials and correct setup before initiating new sampling runs, further minimizing the risk of sample carryover and preventing cross-contamination. According to the Pharmaceutical Inspection Co-operation Scheme (PIC/S) guidelines which provides recommendations for standards in the GMP field, containers and valves should be cleaned using a validated procedure to ensure the absence of any contaminants such as fabrication aids (e.g., lubricants) or microbiological contaminants (PIC/S, 2023).

Efforts need to be taken to reduce the particulates in the different tubing sets used throughout the set-up. To cater to higher frequency sampling requirements, we would also expand the rotary valve capabilities, such as using a 24/36-port rotary valve compared to the 12-port rotary valve. This would minimize the need for microtubes being switched out in room environment, reducing the risk of contamination. Lastly, continuing software development could further improve functionalities such as secure access and operation logs, which are critical for maintaining data integrity and traceability, thereby ensuring audit trails and regulatory compliance. (Steinborn, 2004). Safeguards may be incorporated to make it difficult or impossible for accidental user errors to occur, such as by automating checks and using various error-detection methods. Ultimately, equipment design, operator training, and process control must align with a robust contamination control strategy (CCS) and Quality Risk Management (QRM) principles to ensure sampling activities do not jeopardize product sterility or introduce product variability.

## 5 Conclusion

We have presented the Auto-CeSS as an automated at-line sampling platform, with a minimum sampling volume of 30 μL, making it suitable for the small-volume sampling requirements of scaled-down microbioreactors. Its agnostic capability allows it to be integrated with multiple types of bioreactors. Our work has successfully interfaced the system with the Breez microbioreactor and G-Rex 10M-CS for the purpose of daily sampling of cell-free medium to analyze the metabolic profiles of T cell cultures. The results of key metabolites such as glucose and lactate indicated that the Auto-CeSS is capable of generating samples with high quality, consistency and similarity to manual methods of sampling, with the exception of ammonia, whereby the deviation in ammonia levels was caused by headspace evaporation.

Auto-CeSS serves as an automated tool to streamline key bottlenecks of in-process sampling during CAR T cell manufacturing, such as solving manpower and logistical requirements while minimizing sterility risks in cell therapy manufacturing. Furthermore, in the cell therapy manufacturing industry, development of automated closed systems in conjunction with high throughput bioreactor systems for parallel manufacturing would be highly sought after due to the opportunity to scale out to meet patient demands.

Future work can be done to integrate Auto-CeSS with optical PATs such as Raman spectroscopy and UV absorbance spectroscopy. This would allow samples pulled by Auto-CeSS to be measured in a non-destructive, label free manner, thus potentially enabling an on-line sampling system with real-time closed-loop feedback control for process optimization, while at the same time conserving limited samples and eliminating sterility risks. Predictive modeling can also be applied to get a better sensing of cell metabolism through the automatically collected samples. This could ultimately result in the implementation of adaptive manufacturing processes with simplified workflows via better analytics and performance-based control of cell growth parameters, which could potentially minimize variability and result in the production of cell therapies with improved yield and quality (Wiley Online Library, 2024; Liu et al., 2021). A potential end goal of the Auto-CeSS is to become a part of the GMP processes of cell therapy manufacturing.

## Data Availability

The original contributions presented in the study are included in the article/Supplementary Material, further inquiries can be directed to the corresponding author.
