# Supplementary material for: Automated, aseptic sampling with small-volume capacity from microbioreactors for cell therapy process analysis
Source: Front Bioeng Biotechnol. 2025 Jul 31;13:1612648. doi: 10.3389/fbioe.2025.1612648 (PMC12351187; doi:10.3389/fbioe.2025.1612648)
Supplement: Supplementary file 1 [file Presentation1.pptx]

## Slide 1
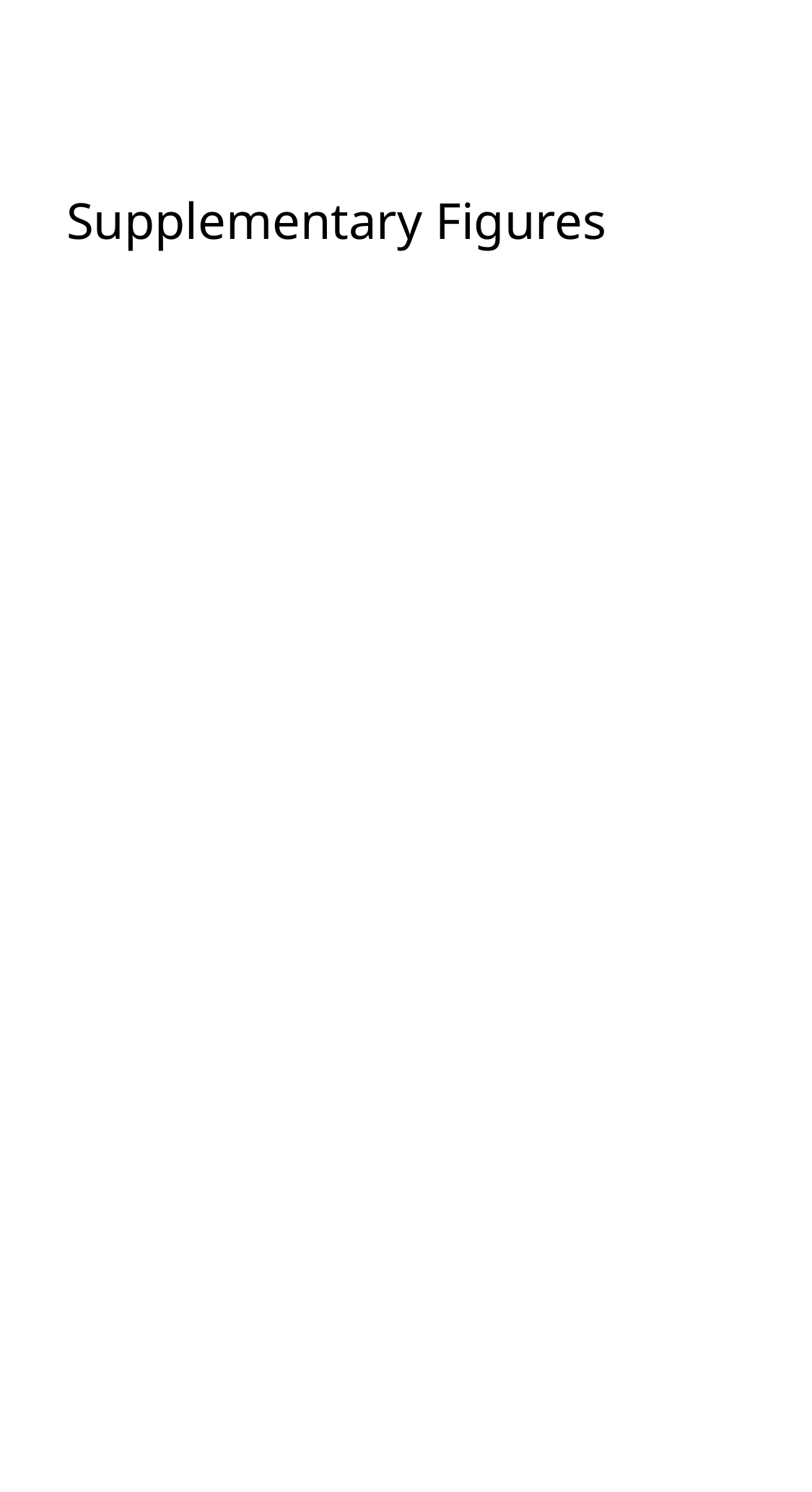

# Supplementary Figures

## Slide 2
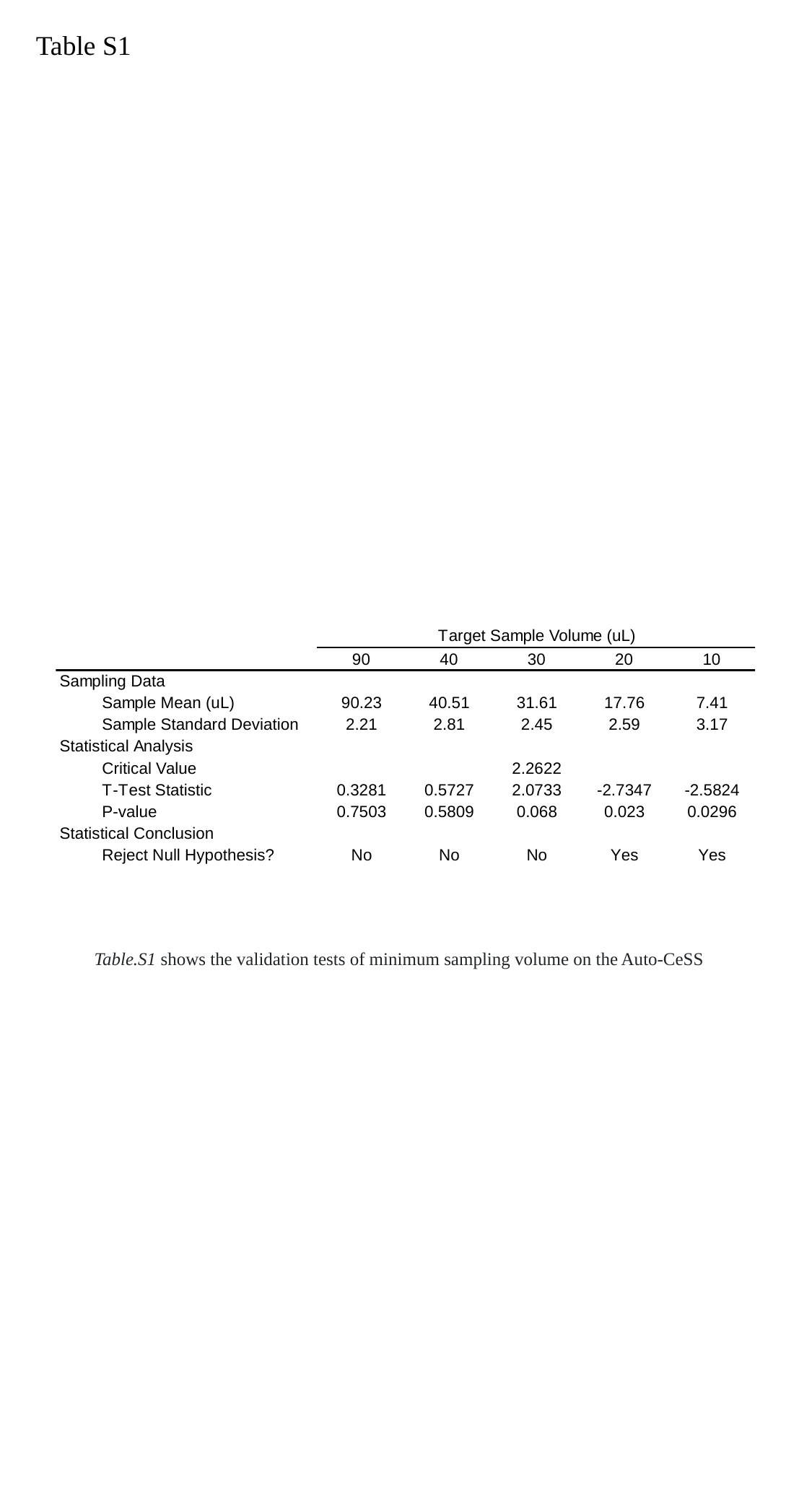

Table S1
Table.S1 shows the validation tests of minimum sampling volume on the Auto-CeSS

## Slide 3
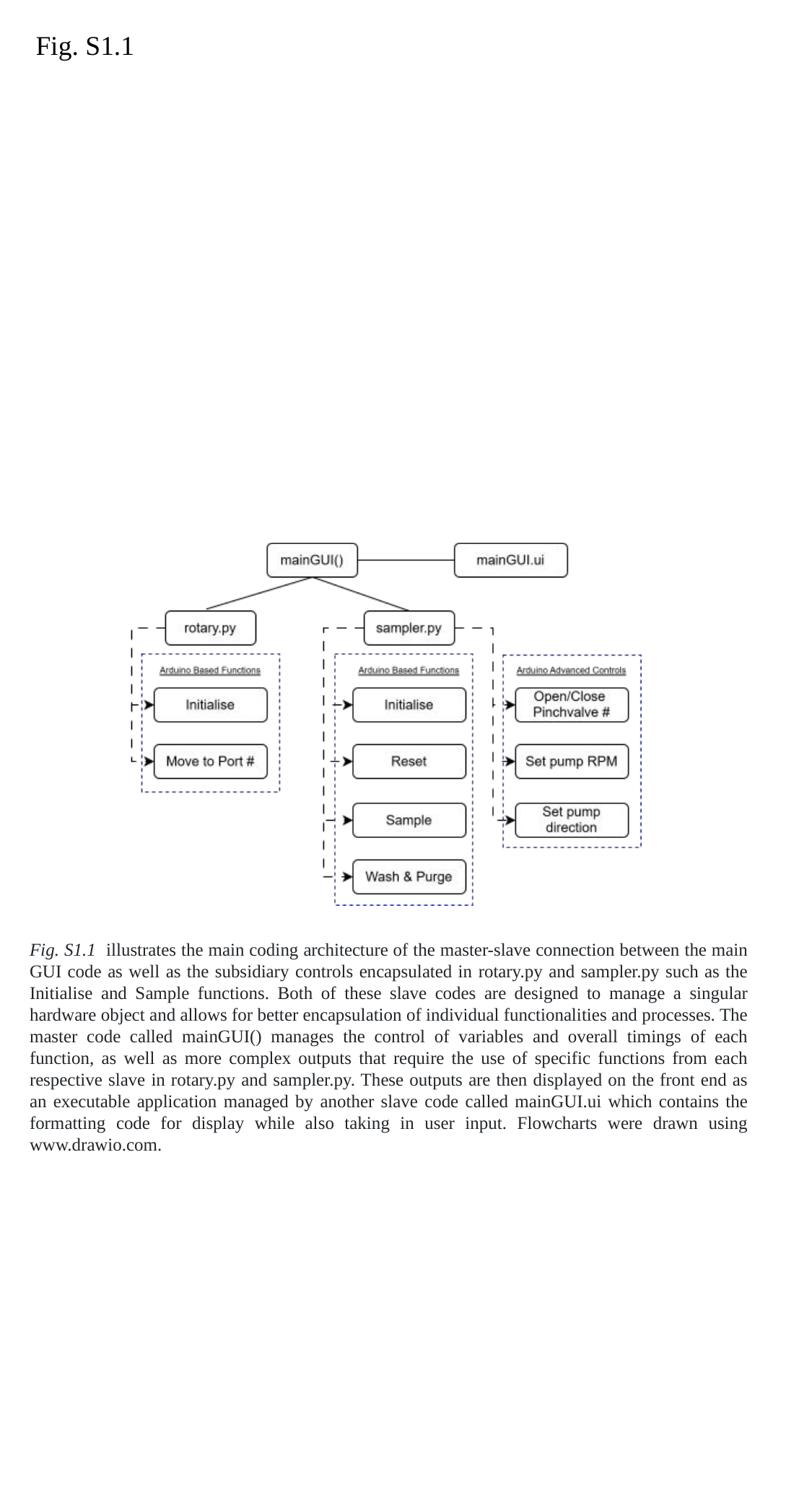

Fig. S1.1
Fig. S1.1 illustrates the main coding architecture of the master-slave connection between the main GUI code as well as the subsidiary controls encapsulated in rotary.py and sampler.py such as the Initialise and Sample functions. Both of these slave codes are designed to manage a singular hardware object and allows for better encapsulation of individual functionalities and processes. The master code called mainGUI() manages the control of variables and overall timings of each function, as well as more complex outputs that require the use of specific functions from each respective slave in rotary.py and sampler.py. These outputs are then displayed on the front end as an executable application managed by another slave code called mainGUI.ui which contains the formatting code for display while also taking in user input. Flowcharts were drawn using www.drawio.com.

## Slide 4
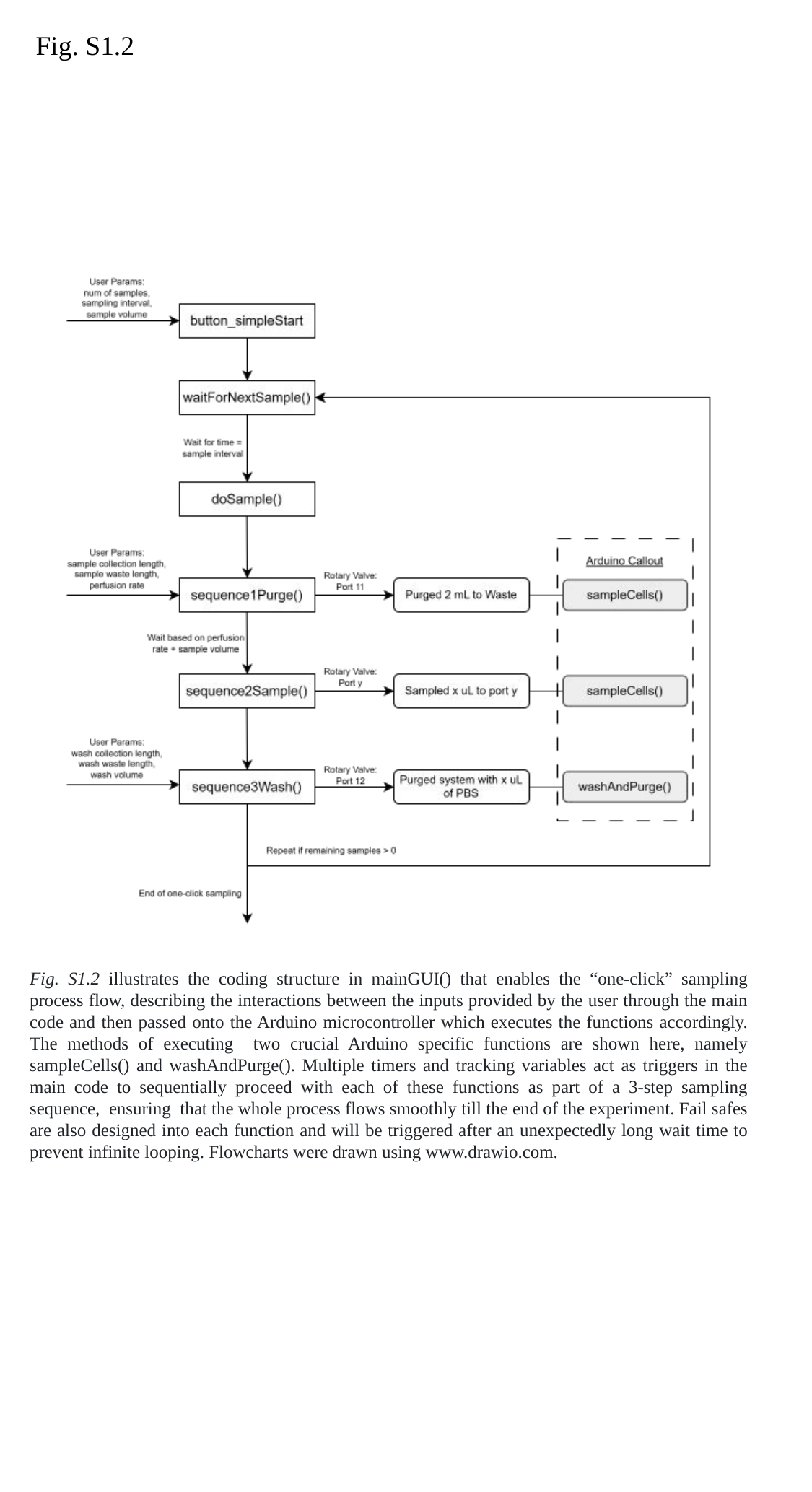

Fig. S1.2
Fig. S1.2 illustrates the coding structure in mainGUI() that enables the “one-click” sampling process flow, describing the interactions between the inputs provided by the user through the main code and then passed onto the Arduino microcontroller which executes the functions accordingly. The methods of executing two crucial Arduino specific functions are shown here, namely sampleCells() and washAndPurge(). Multiple timers and tracking variables act as triggers in the main code to sequentially proceed with each of these functions as part of a 3-step sampling sequence, ensuring that the whole process flows smoothly till the end of the experiment. Fail safes are also designed into each function and will be triggered after an unexpectedly long wait time to prevent infinite looping. Flowcharts were drawn using www.drawio.com.

## Slide 5
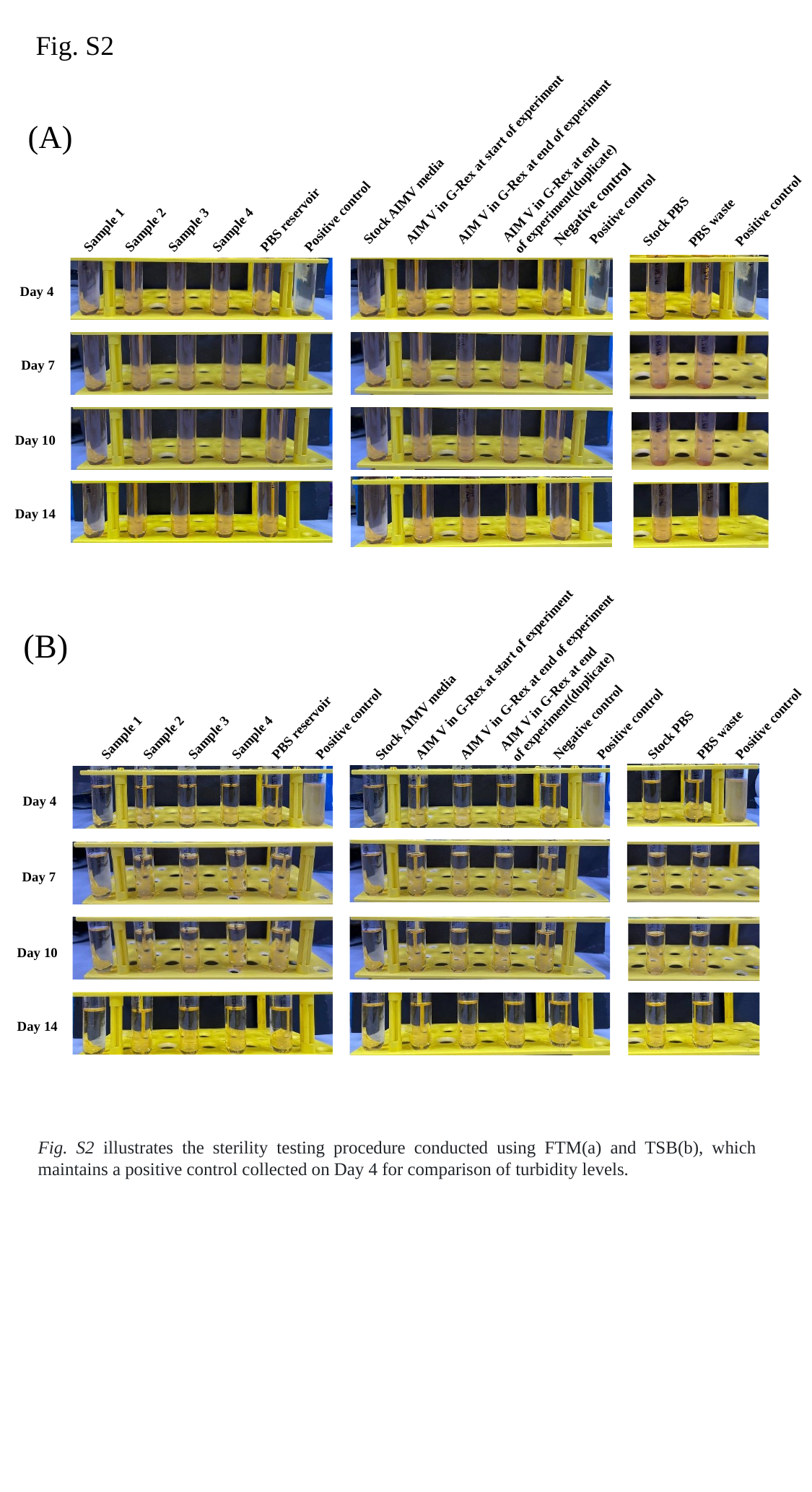

AIM V in G-Rex at end of experiment
AIM V in G-Rex at start of experiment
AIM V in G-Rex at end of experiment(duplicate)
Negative control
Stock AIMV media
Positive control
(A)
Positive control
PBS reservoir
Sample 1
Sample 4
Sample 2
Sample 3
Stock PBS
Positive control
PBS waste
Day 4
Day 7
Day 10
Day 14
Fig. S2
AIM V in G-Rex at start of experiment
AIM V in G-Rex at end of experiment
AIM V in G-Rex at end of experiment(duplicate)
Stock AIMV media
Negative control
Positive control
(B)
Stock PBS
PBS waste
Positive control
Positive control
PBS reservoir
Sample 1
Sample 2
Sample 3
Sample 4
Day 4
Day 7
Day 10
Day 14
Fig. S2 illustrates the sterility testing procedure conducted using FTM(a) and TSB(b), which maintains a positive control collected on Day 4 for comparison of turbidity levels.

## Slide 6
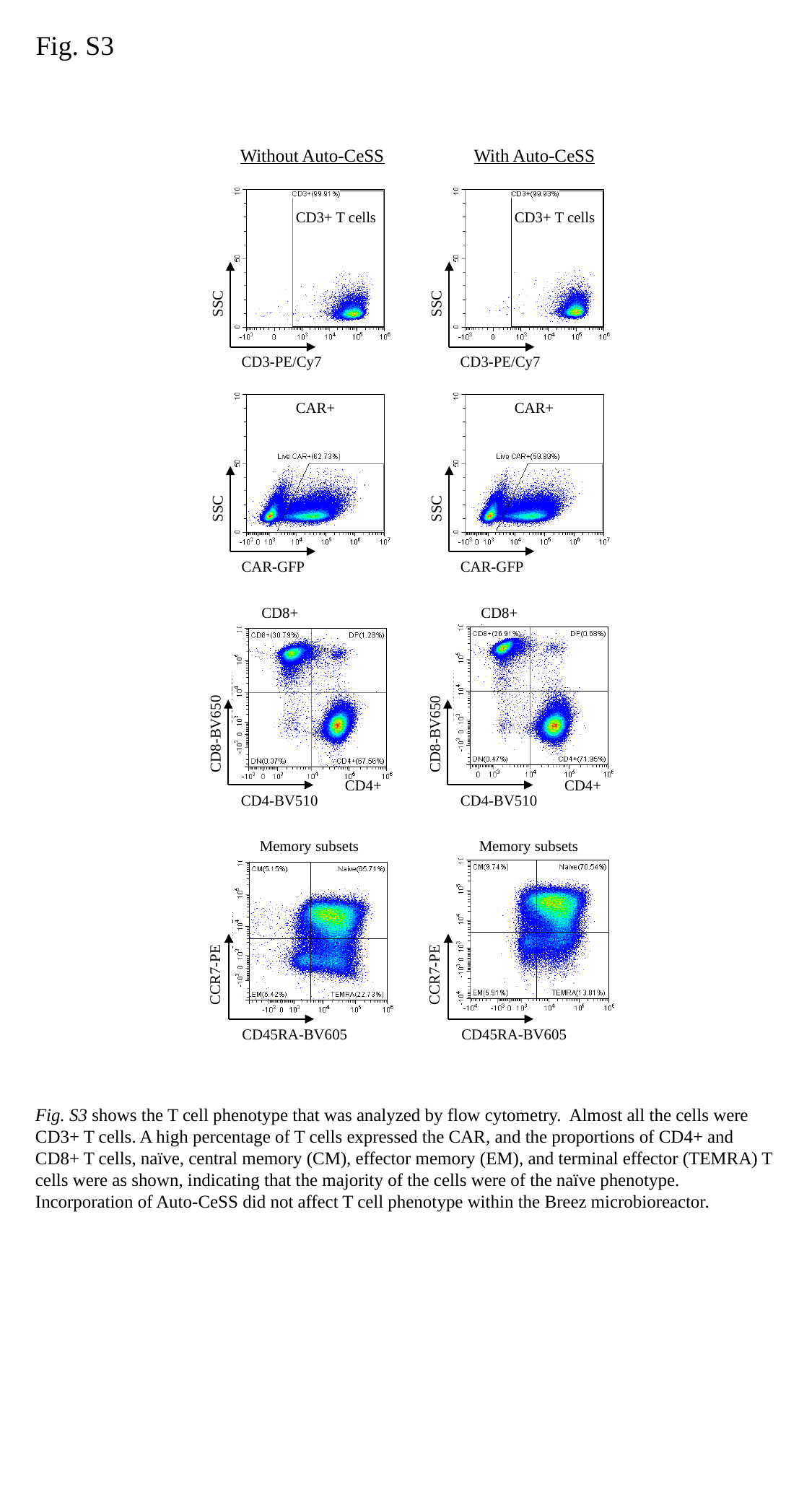

Fig. S3
Without Auto-CeSS
With Auto-CeSS
CD3+ T cells
SSC
CD3-PE/Cy7
CD3+ T cells
SSC
CD3-PE/Cy7
CAR+
SSC
CAR-GFP
CAR+
SSC
CAR-GFP
CD8+
CD8-BV650
CD4-BV510
CD4+
CD8+
CD8-BV650
CD4-BV510
CD4+
Memory subsets
CCR7-PE
CD45RA-BV605
Memory subsets
CCR7-PE
CD45RA-BV605
Fig. S3 shows the T cell phenotype that was analyzed by flow cytometry. Almost all the cells were CD3+ T cells. A high percentage of T cells expressed the CAR, and the proportions of CD4+ and CD8+ T cells, naïve, central memory (CM), effector memory (EM), and terminal effector (TEMRA) T cells were as shown, indicating that the majority of the cells were of the naïve phenotype. Incorporation of Auto-CeSS did not affect T cell phenotype within the Breez microbioreactor.
